# The COVID-19 Pandemic and Professional Nursing Practice in the Context of Hospitals

**DOI:** 10.3390/healthcare10020326

**Published:** 2022-02-09

**Authors:** Olga Maria Pimenta Lopes Ribeiro, Letícia de Lima Trindade, André Filipe Morais Pinto Novo, Carla Gomes da Rocha, Clemente Neves Sousa, Paulo João Figueiredo Cabral Teles, Ana Catarina Rodrigues da Silva Reis, Alessandro Rodrigues Perondi, Karen Cristina Kades Andrigue, Soraia Cristina de Abreu Pereira, Paula Cristina da Silva Leite, João Miguel Almeida Ventura-Silva

**Affiliations:** 1 Nursing School of Porto, Center for Health Technology and Services Research, 4200-072 Porto, Portugal; clemente.n.s@gmail.com; 2 Nursing Departament, Santa Catarina State University, Chapecó-Santa Catarina 89802-111, Brazil; letrindade@hotmail.com; 3 Instituto Politécnico de Bragança, Center for Health Technology and Services Research, 5300-253 Porto, Portugal; andre@ipb.pt; 4 Institute of Health, School of Health Sciences, HES-SO Valais-Wallis, CH-1950 Sion, Switzerland; carla.gomesdarocha@hevs.ch; 5 School of Economics, University of Porto, 4200-465 Porto, Portugal; pteles@fep.up.pt; 6 Centro Hospitalar, Universitário de São João, 4200–319 Porto, Portugal; anacatarinareis@outlook.com (A.C.R.d.S.R.); enf.joao.ventura@gmail.com (J.M.A.V.-S.); 7 Departament of Nursing, University of Paraná, Francicso Beltrão 85601-000, Brazil; alessandroperondi@prof.unipar.br; 8 Nursing Departament, Regional Community University of Chapecó, Chapecó-Santa Catarina 89802-000, Brazil; karenandrigue@unochapeco.edu.br; 9 ACES Entre Douro e Vouga I–Feira/Arouca, North Region Health Administration, 4000-447 Porto, Portugal; pereirasoraia87@gmail.com (S.C.d.A.P.); pleite@arsnorte.min-saude.pt (P.C.d.S.L.)

**Keywords:** coronavirus infection, hospitals, pandemic, quality of health care, work environment

## Abstract

The COVID-19 pandemic has imposed challenges to health systems and institutions, which had to quickly create conditions to meet the growing health needs of the population. Thus, this study aimed to assess the impact of COVID-19 on professional nursing practice environments and to identify the variables that affected their quality. Quantitative, observational study, conducted in 16 Portuguese hospitals, with 1575 nurses. Data were collected using a questionnaire and participants responded to two different moments in time: the pre-pandemic period and after the fourth critical period of COVID-19. The pandemic had a positive impact on the Structure and Outcome components, and a negative trend in the Process component. The variables associated with the qualification of the components and their dimensions were predominantly: work context, the exercise of functions in areas of assistance to COVID-19 patients, length of professional experience and length of experience in the service. The investment in professional practice environments impacted the improvement of organizational factors, supporting the development of nurses’ work towards the quality of care. However, it is necessary to invest in nurses’ participation, involvement and professional qualifications, which are aspects strongly dependent on the institutions’ management strategies.

## 1. Introduction

In the last decade, professional practice environments and their impact on professionals, clients, and institutions have been one of the main research areas [1,2,3,4]. However, in the context of a pandemic, it becomes crucial to quickly identify the weakest areas and define strategies for improvement.

Despite the high pressure COVID-19 placed on various health systems and their professionals [5], the disease caused by the Severe Acute Respiratory Syndrome Coronavirus 2 (SARS-CoV2), which quickly spread worldwide, having determined that in March 2020, the World Health Organization (WHO) would declare a pandemic [6], brought relevance and visibility to the nurses’ role in health care, as well as in the management area, particularly in the rapid restructuring of services [7].

It is known that nurses were asked to work in highly complex and uncertain environments, where all individual resources had to be mobilized to quickly adapt to the numerous changes imposed by the pandemic [5,8]. Along with the need for reorganization of health services [7], one study highlights that the current pandemic has triggered psychological experiences shaped by the structural conditions of the health system and its institutions, with its repercussions on professional practice [9].

Representing the main workforce in the health area, nursing professionals were on the front lines from the beginning of the pandemic, and experienced a negative impact on their mental health [5,10], whose severity was aggravated by the poor working conditions, which, in many situations, were present before the pandemic [7]. In fact, over the last decade, nurses have been paying attention to the need to improve the nursing professional practice environments, but this investment has not been sufficiently noticeable. As a consequence, the COVID-19 pandemic aggravated some of the difficulties that these professionals were already facing, particularly in hospital settings [5,11].

In addition, nurses represent a workforce that is difficult to replace. The threat to their health and well-being may lead to negative repercussions on themselves and the care they provide [5]. Thus, to avoid the collapse of services and the nursing workforce, the challenge involves ensuring practice environments that are more favorable to the quality of care and nurses’ satisfaction, commitment, and involvement in the continuum of facing the pandemic and after it. Authors [12] found that the commitment to the organization was the strongest predictor of professional retention, which is particularly relevant in the context of the current pandemic, knowing that the retention of qualified and experienced nurses is critical for the institutions’ management in all countries.

In Portugal, the pandemic caused by the spread of SARS-CoV2 has already infected 1,389,646 people and led to the death of 18,955 others since its emergence in February 2020 until December 2021 [13]. Given the scarce resources and previous weaknesses in the Portuguese health system, the pandemic has caused immense pressure on it and its professionals, requiring investment in professional practice environments to ensure the safety and quality of care [14], as well as the professionals’ well-being.

After the first critical period of the pandemic in this country, several hospitals have developed COVID-19 contingency plans, with the sequential definition of services to be opened in case of an increase in the number of hospitalisations in the different care areas, allowing for an adequate response to the growing care needs, as well as to the need to adopt preventive measures to limit SARS-CoV-2 transmission. Within the scope of logistical and structural restructuring, in the most recent critical periods of the pandemic, the fact that the top management bodies of some institutions anticipated the need for reorganisation, creating specific care areas for patients with COVID-19 and for patients awaiting test results, has been essential [14].

However, with the increase in the number of beds and, in some situations, the opening of new services becomes necessary to reinforce the healthcare teams. The problem is that, given the difficulties in hiring health professionals, namely nurses, these professionals have been moving between services and changing their previous functions. Even in adopting these strategies, there are many settings in which the high number of patients assigned to each nurse, the complexity of care and the high workloads make it difficult to meet all care needs [8,11,14].

Although the difficult working conditions could compromise the values of nursing international studies proved that this did not occur, and that the nurses’ motivation and spirit of their mission were essential to overcome the difficulties [15]. However, after months of physical and emotional wear, witnessing the clinical worsening and even the death of many patients, several authors highlighted the need to study the intensity of the pandemic’s impact on the professionals [15] and their work environment [16].

In recent years, there is growing evidence that the common predictive factors for nurses’ well-being, quality and safety of care refer to the dimensions of professional practice environments [4,17], reinforcing the importance of assessing and identifying opportunities for improvement. To this end, it is essential to use assessment tools adjusted to the work contexts and the reality of professional practice. The instrument developed and validated in Portugal allows assessing three essential components: the Structure, which refers to the organizational factors that allow nurses to develop their work, as well as the conditions under which care is provided; the Process, which refers to the factors related to the performance of activities inherent to the design and provision of nursing care; and the Outcome, which assesses the desirable or undesirable changes regarding care, patients, and nurses [18].

Given the evidence that the professional practice environment is essential for the quality of care and that the components Structure, Process, and Outcome are essential to ensure it [19,20], the hypotheses of our study are: COVID-19 has an unequal impact on the components of nursing professional practice environments; the professional variables (e.g., work context, exercise of functions in areas of care for patients with COVID-19, length of professional experience and length of professional experience in the service) affect the qualification of the components/dimensions of nursing professional practice environments.

The relevance of this study is based on the importance of knowing the impact of COVID-19 and exploring the factors that may have positive or negative effects on nursing professional practice environments, thus providing information to institutions, nurses and professional representative bodies so as to support changes that allow for a quality and efficient response to the care needs, which have been greatly aggravated by the pandemic. In addition, the authors are motivated to promote more positive professional nursing practice environments, which ensure the quality and safety of care and the well-being of nurses, who have been absolutely crucial in the fight against the pandemic.

### Objective

The COVID-19 pandemic imposed several challenges to health systems and professionals, thus generating the need to invest in the improvement of nursing professional practice environments. In this context, the identification of the most fragile areas and the definition of improvement strategies will allow addressing some of the most emerging gaps. Thus, the objectives of this study were to assess the impact of COVID-19 on nursing professional practice environments and to identify the variables that affected their quality.

## 2. Materials and Methods

### 2.1. Design

The quantitative, observational study, presented with the support of the tool Strengthening the Reporting of Observational Studies in Epidemiology (STROBE^®^).

### 2.2. Participants

Using a non-probability convenience sampling technique, 1575 registered nurses from 16 hospital institutions in mainland Portugal participated in the study, which corresponded to a participation rate ranging between 26 and 32%. The inclusion criteria were defined as being a nurse or a specialist nurse and working in the Departments of Medicine, Surgery, Emergency and Intensive Care, Psychiatry and Mental Health, and Women and Children. All absent professionals due to leave or holiday during the data collection period were excluded.

### 2.3. Instruments

As a data collection instrument, we used a self-completion questionnaire composed of two parts: sociodemographic and professional characterization of the participants (gender, age, marital status, academic background, professional status, area of specialty, work context and length of professional experience) and Scale for the Environments Evaluation of Professional Nursing Practice (SEE-Nursing Practice) [18].

The SEE-Nursing Practice, which was built and validated in 2020, is composed of three subscales: the SEE-Nursing Practice—Structure (with 43 items divided into six dimensions), the SEE-Nursing Practice—Process (with 37 items divided into six dimensions) and the SEE-Nursing Practice—Outcome (with 13 items divided into two dimensions). Each item is answered on a Likert-type scale with five options, where one corresponds to “never”, two “rarely”, three “sometimes”, four “often” and five “always” [18].

### 2.4. Data Collection

Data were collected through the online completion of the questionnaire. In relation to the items of the SEE-Nursing Practice [18], participants were asked to respond to two different moments in time: pre-pandemic moment and “current” moment, which, in this study, was after the fourth critical period of the COVID-19 pandemic in Portugal. Following the parameters used in the country (new cases, deaths, admissions to hospital wards and intensive care units, transmissibility index and incidence of SARS-CoV-2 infections), the critical period was considered to be that in which there was a higher number of cases, a higher number of patients hospitalised for COVID-19, in hospital wards and intensive care units and a higher number of deaths, with a subsequent decrease in all these parameters [21]. Thus, data collection took place from 15 August to 15 October 2021. It should be noted that, although the two months of data collection corresponded to the recovery time of the fourth critical period, a 5th critical period was already imminent in Portugal, which occurred in November 2021 [21].

In order to collect data, as previously authorised by the ethics committees, an email was sent to the nurse managers of each hospital institution with information about the study and a link for participants to access the questionnaire. Subsequently, the nurse managers transferred the link to the institutional email of all nurses of the services included in the study.

### 2.5. Data Analysis

Data were processed using the IBM Statistical Package for the Social Sciences (SPSS), version 26.0 (Armonk, New York, USA), using descriptive and inferential statistics. When analyzing the results, the higher the score in the SEE-Nursing Practice, the more favorable was the environment of professional nursing practice to the quality of care. For the analysis, the following criteria were established: score < 35%—nursing professional practice environment component slightly favorable to the quality of care; a score between 35% and 55%—nursing professional practice environment component moderately favorable to the quality of care; score between 55% and 75%—nursing professional practice environment component favorable to the quality of care; and, finally, score > 75%—nursing professional practice environment component highly favorable to the quality of care.

The Cronbach’s alpha values of the SEE-Nursing Practice components, in relation to the pre-pandemic and post-fourth period COVID-19 data, were 0.959 and 0.957 in the Structure component, 0.933 and 0.936 in the Process component, and 0.933 and 0.926 in the Outcome component, respectively. Such values were overall higher than in the previous study [18].

It should be noted that the SEE-Nursing Practice, in addition to its good psychometric properties, may provide useful information on the dimensions of the best and worst scored nursing professional practice environments, thus allowing for the identification of improvement needs.

At the beginning of the statistical analysis, using the Shapiro–Wilk and Lilliefors tests, normality was rejected for all dimensions and subscales. Consequently, for the variable “nursing professional practice environments”, comparisons between the pre-pandemic moment and after the fourth critical period of COVID-19 were based on the Wilcoxon Test (paired samples). The significance level adopted was 5%. Next, to identify the variables that affected the nursing professional practice environments and in what way, the multiple linear regression model fitted by OLS with stepwise selection was used. The explanatory variables of the model were the characterization attributes, i.e., gender, age, marital status, education, professional status, area of specialization, work context, and length of professional experience. For the selection of explanatory variables, the variables whose estimated parameters had a *p*-value lower than the adopted significance level of 0.05 were retained in the model, showing that they are statistically significant and that the respective variables have an impact on nursing professional practice environments.

### 2.6. Ethical Approval and Informed Consent

The study was initially approved by a health ethics committee under number 104/21 and, subsequently, by the ethics committees of the respective hospital institutions, as well as authorized by the management boards of these institutions. The informed consent form was submitted online, with a clarification page about the study. The completion of the questionnaire was only possible after the nurse agreed to participate in the research. Confidentiality and anonymity were ensured in the use and dissemination of the obtained data.

## 3. Results

### 3.1. Characterisation of the Participants

A total of 1575 nurses participated in the study, whose sociodemographic and professional characteristics are shown in Table 1.

### 3.2. Environments of Professional Nursing Practice

Regarding the environments of professional nursing practice, the results are shown in Table 2.

In the Structure component, it was found that after the fourth critical period of COVID-19, the average percentage in all dimensions and in the subscale itself was higher. In the Process component, except for the dimensions “collaboration and teamwork” and “interdependent practices in professional practice”, in all the others, the percentage was lower after the fourth critical period of COVID-19. Finally, in the Outcome component, after the fourth critical period of COVID-19, the average percentage in both dimensions and in the subscale itself was higher.

### 3.3. Sociodemographic and Professional Variables and the Environments of Professional Nursing Practice

In order to identify the variables that affected the environments of professional nursing practice, a regression model was fitted, whose results are explained in Table 3.

With regard to the Structure component, before COVID-19, female nursing professionals, divorced and working in the Women and Children’s Department showed a higher mean score. Nurses from the Surgery Department evidenced a lower mean score. The longer the time of professional experience, the higher the mean score in the Structure component. On the other hand, nurses with a longer tenure of professional experience in the service showed a lower mean score in this component.

After the fourth critical period of COVID-19, nurses from the Women and Children’s Department, the Psychiatry and Mental Health Department and, subsequently, nurses from the Medicine Department showed higher mean scores. The longer the length of professional experience, the higher the mean score. On the other hand, married nurses, with longer time of professional experience in the service and working in areas of care to patients with COVID-19 showed lower mean scores in this component.

With regard to the Process component, before COVID-19, female nursing professionals working in the Women and Children’s Department showed a higher mean score. On the other hand, nurses from the Emergency and Intensive Care Department, followed by the Surgery Department, evidenced lower mean scores.

After the fourth critical period of COVID-19, female nursing professionals and working in the Women and Children’s Department had the highest mean score, followed by nurses in the Medicine Department and Psychiatry and Mental Health Department, who had the third-highest mean score.

With regard to the Outcome component, before COVID-19, female and older nursing professionals had a higher mean score. Married nursing professionals working in the Emergency and Intensive Care Department and with longer time of professional exercise in the service, showed a lower mean score.

After the fourth critical period of COVID-19, married nursing professionals from the Emergency and Intensive Care Department and the Surgery Department, and working in areas of care for patients with COVID-19, evidenced lower mean scores.

Due to the importance, after the fourth critical period of COVID-19, of identifying the variables that affected the various dimensions of nursing professional practice environments, regression models were again used, whose results are shown in Table 4, referring to the Structure, Process and Outcome components.

In relation to the Structure component, in dimension “People management and service leadership”, the mean score was higher among divorced nurses and among those who had been working longer. On the other hand, nurses who worked in the Surgery Department had a lower mean score.

In relation to the dimension, “Physical environment and conditions for appropriate service running”, older nurses and nurses with a bachelor’s degree showed a lower mean score. On the other hand, single nurses working in the Medicine Department in areas of care to patients with COVID-19 and with a longer period of professional experience showed a higher mean score.

With regard to the dimension “Nurses’ participation and involvement in the institution’s policies, strategies and management”, married nurses and nurses working in the Emergency and Intensive Care Department showed a lower mean score. On the other hand, specialist nurses and those working in areas of assistance to patients with COVID-19 showed a higher mean score.

In relation to the dimension “Institutional policy for professional qualification”, nurses working in the Emergency and Intensive Care Department and in the Surgery Department presented lower mean scores. On the other hand, nurses working in the Psychiatry and Mental Health Department had higher mean scores.

With regard to the dimension, “organisation and guidance of nursing practice”, nurses working in the Women and Children’s Department had the highest mean score, followed by nurses working in the Medicine Department who had the second-highest mean score.

In the dimension, “Quality and safety of nursing care”, female nurses, those who worked in the Women and Children’s Department and those who had been working for an extended period of time showed a higher mean score. On the other hand, nurses with more professional experience showed a lower mean score.

Within the Process component (Table 4), regarding the dimension, “Collaboration and teamwork”, female nursing professionals, those who worked in the Medicine Department, in areas of care for patients with COVID-19 and those who had been working longer, showed higher mean scores.

In relation to the dimension “Strategies for ensuring quality in professional practice”, female nursing professionals and those working in the Medicine Department showed higher mean scores.

With regard to the dimension “Autonomous practices in professional practice”, nurses working in the Women and Children’s Department had the highest mean score. On the other hand, specialist nurses and nurses working in the Emergency and Intensive Care Department and in the Surgery Department had lower mean scores.

In relation to the dimension, “Care planning, evaluation, and continuity”, older nurses, nurses who worked in areas of care to patients with COVID-19 and those who worked in the Women and Children’s Department and in the Medicine Department had higher mean scores. On the other hand, married nurses, specialists and nurses with a longer period of professional experience in the service showed a lower mean score.

With regard to the dimension “Theoretical and legal support of professional practice”, nurses from the Surgery Department had the lowest mean score, followed by nurses from the Emergency and Intensive care Department, who had the second-lowest mean score.

In relation to the dimension “Interdependent practices in professional practice”, single nurses and older nurses showed a lower mean score. On the other hand, nurses practicing as specialists, working in the Women and Children’s Department and in areas of care to patients with COVID-19, and with a longer period of professional experience, presented the highest mean score.

Within the Outcome component (Table 4), regarding the dimension “Systematic assessment of nursing care and indicators”, single nurses and nurses with a bachelor’s degree presented the highest mean score. On the other hand, nurses working in the Emergency and Intensive Care Department gave the lowest mean score, followed by nurses from the Surgery Department who had the second-lowest mean score.

In the dimension, “Systematic assessment of nurses’ performance and supervision”, nurses with higher age presented a higher mean score. On the other hand, married nurses, working in areas of care to patients with COVID-19 in the Women and Children’s Department and in the Emergency and Intensive Care Department had lower mean scores.

## 4. Discussion

One of the main objectives of this study was to assess the impact of COVID-19 on professional nursing practice environments, and the results show a positive impact on the Structure and Outcome components and a negative trend on the Process component.

In a pandemic context, the investment in structural conditions, such as the nurse manager’s leadership, the availability of material resources, the adequacy of the number of patients per nurse, the admission/hiring of more nursing professionals, the participation and involvement in decision-making, and the timely support to nurses were essential for the teams to remain focused on the quality and safety of care [22]. In our study, the mentioned strategies justify the increase in the mean score of the Structure component. It should be noted that the dimension “nurses’ participation and involvement in the institution’s policies, strategies and management” had the lowest mean percentage in both moments. Before the pandemic, the nurses’ participation in hospital affairs was the worst scored factor [1]. In a study conducted in Brazil about the work environments in hospitals during the pandemic, the nurses’ unfavorable aspects included the low participation in decision-making [16].

In addition to the investment in structural conditions, from the perspective of some authors, only with the systematic assessment of care and nursing indicators will be possible to ensure the success of the processes implemented in response to the challenges imposed by the pandemic, which also justifies the higher score obtained in the Outcome component [22].

The difficulty in investing in autonomous practices, in acting in line with the theoretical and legal frameworks of professional practice and in planning, assessing and ensuring the continuity of care had a negative impact on the Process component. The severity of the patients’ clinical condition, the conflict between the duty of care and the high risk of infection [23,24], the increased workload, the difficulty for nurses to know the patients’ previous condition due to the restriction of family visits have hampered, in the context of a pandemic, a nursing practice focused on the health/disease transition experienced by the person, which effectively represents the essence of nursing [25].

In relation to the variables associated with the Structure component, working in the Women and Children’s Department and presenting longer professional practice resulted in a higher mean score before the pandemic and after the fourth critical period of COVID-19. The Women and Children’s Department services are also living spaces, where, in addition to the disease, nursing professionals experience birth. Furthermore, in the context of COVID-19, children recorded lower prevalence and mortality from SARS-CoV2 [26,27].

On the other hand, the increased time working in the service and the performance of functions in care areas to patients with COVID-19 culminated in a lower mean score in the Structure component, showing that despite the investment in these contexts [7,22], the demands were higher. The increased time spent working in services that already had significant problems, such as the high workload and pace of work, as well as the lack of human and material resources [28], may have determined a lower mean score in the Structure component.

With regard to the dimensions of the Structure component after the fourth critical period of COVID-19, nurses with significant professional experience presented higher mean scores in the dimensions “people management and service leadership”, “physical environment and conditions for appropriate service running” and “quality and safety of nursing care”. Work for an extended period of time often represents having had difficult professional experiences, which, in the context of a pandemic, facilitates the recognition of the work performed by the institutions’ middle and senior managers in the creation of conditions to respond with quality and efficiency to the needs arising from the pandemic, ensuring the quality and safety of care [7,29]. Nurses working in areas caring for patients with COVID-19 showed higher mean scores in the dimensions “physical environment and conditions for appropriate service running” and “nurses’ participation and involvement in the institution’s policies, strategies and management”. During the pandemic, the investment in these dimensions, particularly in services with patients with COVID-19 [22], justifies the obtained results. Although in the first months of the pandemic there were frequent reports of difficult working conditions [30], the results of this study show that over the months working in areas caring for patients with COVID-19, there was an improvement in working conditions, as well as more opportunities to participate in the institution’s decisions and policies. 

This justifies the fact that even in pandemic circumstances, nurses maintained moderate to high job satisfaction [15]. Nurses who worked in services of the Medicine Department had higher mean scores in the dimensions “physical environment and conditions for appropriate service running” and “organization and guidance of nursing practice”. In Portugal, the reorganization of the services of the Medicine Department was determinant to the extent that, in many of these settings, the increase in the number of hospitalizations of patients with COVID-19 made it urgent to create conditions to ensure the service running and the organization of nursing practice [31]. 

On the other hand, nurses who worked in the Surgery Department presented lower mean scores in the dimensions “people management and service leadership” and “institutional policy for professional qualification”. In order to keep the surgical activity previously planned, the number of COVID-19 patients admitted to the services of the Surgery Department was low. This made the relevance of people management and leadership by the nurse manager less evident, as well as the existence of a policy for a professional qualification, at a time when the learning needs in relation to caring for patients with COVID-19 were high [22]. Nurses from the Intensive Medicine and Emergency Department showed a lower score in the dimensions “nurses’ participation and involvement in the institution’s policies, strategies and management” and “institutional policy for professional qualification”. Given they were subjected to high levels of demand, it is possible that these nurses considered the opportunities for participation, involvement and professional qualification as insufficient. The concern to keep themselves updated, to support their practice with the best scientific evidence was something clearly expressed by nurses [8]. However, given the daily standards and guidelines, it is understandable that nurses felt an even greater need to develop skills and abilities to ensure the quality and safety of care. In this context, it is up to nursing managers to be attentive to nurses’ needs and requests, defining the strategies that more readily ensure their empowerment [23,32].

The pandemic mostly negatively influenced the Process component, except in the dimensions “collaboration and teamwork” and “interdependent practices in professional practice”. In view of the speed and unpredictability of events, teamwork allowed filling the gaps created by the need and desire to provide person-centered care [22]. Although the crisis generated by COVID-19 may have fostered cohesion, interdependence and teamwork, organizations need to be aware and committed to supporting nurses during and after the pandemic in order to prevent physical and emotional burnout and abandonment of the institution or the profession itself [12].

Before the pandemic and after the fourth critical period of COVID-19, female nursing professionals and those who worked in the Women and Children’s Department presented higher mean scores in this component. The fact that these services had not contemplated the creation of specific units for the care of patients with COVID-19, and the infrequent presence of patients with suspected infection in these contexts, did not require significant changes in the work process, which justifies that the score in this component was higher.

With regard to the variables associated with the *Process* component after the fourth critical period of COVID-19, nurses working in the Medicine Department scored higher in the dimensions “collaboration and teamwork”, “strategies for ensuring quality in professional practice” and “care planning, evaluation and continuity”, highlighting, once again, the gains obtained with the reorganization of these services [31]. It should be noted that in Portugal, many of the areas of care to patients with COVID-19 were integrated in the Medicine Department, which made the investment in the aspects included in these dimensions even more relevant. Nurses from the Women and Children’s Department presented higher scores in the dimensions “autonomous practices in professional practice”, “care planning, evaluation and continuity,” and “interdependent practices in professional practice”. Although the autonomous dimension of the nursing profession is relevant in the Women and Children’s Department, studies show that the collegial relationships between team members have effectively improved the work environments [33]. Nurses with more years of professional experience and those who worked in areas caring for patients with COVID-19 presented higher mean scores in the “collaboration and teamwork” and “interdependent practices in professional practice” dimensions. 

In addition to longer professional experience contributing to a more positive work environment [29], the increased workload and complexity of care in services, particularly in patients with COVID-19, made teamwork and the focus on the interdependent dimension of the profession necessary [16]. In a study conducted in Portugal, nurses highlighted that team cohesion was strengthened, emphasizing positive aspects of collaboration and sharing of experiences [8]. A study conducted in another country highlighted the importance of collaborative actions between the medical and nursing teams in caring for patients with COVID-19 [7].

On the other hand, nurses working in the Emergency and Intensive Care Department and the Surgery Department presented lower mean scores in the “autonomous practices in professional practice” and “theoretical and legal support of professional practice” dimensions. The severity of the patients’ clinical condition in these contexts and its resolution is highly related to interdependent interventions that justify the previously described results [23,25]. Specialist nurses showed a lower mean score in the dimensions “autonomous practices in professional practice” and “care planning, evaluation and continuity”. The feeling of powerlessness associated with the difficulty of these professionals to meet all the patients’ needs may have influenced the lower scores in the autonomous practices, which include care planning, evaluation and continuity of care. It should be noted that, in Portugal, during the pandemic, the performance of functions in specialised areas was not always possible, since, given the high workload and the lack of general care nurses, specialist nurses were asked to perform those functions [14].

In the Outcome component, after the fourth critical period of COVID-19, the mean percentage in both dimensions and in the subscale itself was higher. Before COVID-19, as well as after the fourth critical period, nursing professionals working in the Emergency and Intensive Care Department presented lower mean scores in this component. In addition, nurses working in these services scored lower in both dimensions, “systematic assessment of nursing care and indicators” and “systematic assessment of nurses’ performance and supervision”. In hospital institutions, the Emergency and Intensive Care Department are the settings where the number of hospitalizations and the complexity of the patients’ clinical condition has been more evident and worrying throughout the pandemic, which is why the monitoring of outcomes has not always been prioritized [14,23].

The findings of this study allow for the suggestion of specific strategies to institutions, institutional managers and nursing managers that can promote the quality of nursing practice environments. Within the scope of the Structure component, in addition to the provision of human and material resources, investments are suggested in the nurses’ participation and involvement in the policies, strategies and functioning of the institutions, as well as actions promoting professional qualification, which take into account the nurses’ needs and requests. With regard to the Process component, in addition to the promotion of collaborative relationships between the members of the multidisciplinary team, the findings reinforce the need for actions/programmes that foster the effective use of nurses’ specific qualifications and skills, ensuring opportunities for investment in autonomous practices, particularly focused on the people who need care. Supervisory models that ensure support for nurses will also be essential to prevent physical and emotional burnout. 

With regard to the Outcome component, as a way to assess the actions developed, in this specific case, in the fight against the pandemic, the monitoring of indicators related to professionals, patients and the institution itself is relevant. In this direction, the scale used in this research, or other assessment instruments, emerges as possible strategies.

Despite the relevance of the results, this study presents some limitations. First, it is an observational study and limited to the Portuguese context. Although at this moment, the study is being replicated in another country, there are still no results that allow for a comparative study, which, in relation to some variables, made it difficult to discuss the findings more broadly. Secondly, in the data collection, the participants were asked to answer with regard to two distinct moments in time: the pre-pandemic moment and the current moment. Although this was a possibility to understand the impact of the pandemic on some dimensions of the practice environments, we should consider the risk of response bias. The online data collection may also be considered as a limitation, as it may have hindered the access and adherence of a more significant number of participants. However, given the restricted circulation in hospital institutions due to COVID-19, the use of online data collection was the possible strategy to ensure the participation of nurses from hospitals in different regions of the country.

However, the study presents findings and suggestions to qualify the Structure, Process and Outcome components of nursing professional practice environments, with positive repercussions on the well-being of nursing professionals and the quality and safety of the care provided.

## 5. Conclusions

The pandemic positively impacted the Structure and Outcome components and a negative trend in the Process component of nursing professional practice environments. The variables associated with the qualification of the components and respective dimensions were predominantly work context, the exercise of functions in areas of care for patients with COVID-19, length of professional experience, and length of experience in the service. 

Despite the positive impact of the pandemic on the Structure and Outcome components of the nursing professional practice environments, these remained moderately favourable to the quality of care. On the other hand, even with a negative impact, we confirmed that the Process component remained favourable to the quality of care, showing that, despite the numerous difficulties, nurses maintained a performance consistent with their social mandate. Even so, it is important to strengthen the need to invest in the conditions that ensure the performance of the activities inherent to the design and provision of nursing care, especially in the autonomous area of the profession, which was, in fact, the most penalised area.

The pandemic with COVID-19 revealed weaknesses in nursing professional practice environments but also generated challenges and opportunities. In this context, the participation in the institutions’ policies, the involvement, the creation of conditions for a professional qualification, and the effective recognition of the nurses’ essential role may be determinants in the development of more positive nursing professional practice environments.

The findings of this study indicate aspects that need to be observed in the components Structure, Process, and Outcome, which contribute to qualifying the environments of professional nursing practice, enhancing the workforce, and ensuring the quality and safety of care.

## Figures and Tables

**Table 1:** Sociodemographic and professional characterization of the participants.

Gender *n* (%)	
Female	1239 (78.7)
Male	336 (21.3)
Marital status *n* (%)	
Single	475 (30.2)
Married/non-marital partnership	998 (63.4)
Divorced	97 (6.2)
Widower	5 (0.3)
Age (years) Mean; Median; Std. Dev.	39.7; 39; 9.2
Education *n* (%)	
Bachelor’s degree	1304 (82.8)
Master’s degree	264 (16.8)
Doctoral degree	7 (0.4)
Work Department *n* (%)	
Medicine Department	640 (40.6)
Surgery Department	429 (27.2)
Emergency and Intensive Care Department	329 (20.9)
Psychiatry and Mental Health Department	96 (6.1)
Woman and Child Department	81 (5.1)
Work in areas of care for COVID-19 patients *n* (%)	993 (63.0)
Time in areas of care for COVID-19 patients (months) Mean; Median; Std. Dev.	8.4; 7; 5.1
Professional category *n* (%)	
Nurse	982 (62.3)
Specialist nurse	593 (37.7)
Time of professional practice (years) Mean; Median; Std. Dev.	16.7; 15; 9.3
Time of professional practice in the service (years) Mean; Median; Std. Dev.	9.4; 7; 8.1
Nurses with Nursing Specialization *n* (%)	635 (40.3)
Nursing specialization area *n* (%)	
Medical-Surgical	220 (34.6)
Rehabilitation	204 (32.1)
Mental and Psychiatric Health	75 (11.8)
Child and Paediatric Health	43 (6.8)
Maternal and Obstetric Health	42 (6.6)
Community	51 (8.1)

Std. Dev.: Standard Deviation.

**Table 2:** Average percentages of the components and dimensions of nursing professional practice environments at the pre-COVID-19 moment and after the fourth critical period of COVID-19.

Components/Dimensions	Pre-COVID-19		After the Fourth Critical Period of COVID-19		
	Mean	Std. Dev.	Mean	Std. Dev.	*p-*Values *
STRUCTURE Component					
Dim 1—People management and service leadership	58.4	18.6	60.6	18.2	<0.001
Dim 2—Physical environment and conditions for appropriate service running	54.5	15.0	54.8	15.5	0.813
Dim 3—Nurses’ participation and involvement in the institution’s policies, strategies and management	43.3	17.1	45.6	18.0	<0.001
Dim 4—Institutional policy for professional qualification	44.9	19.0	46.1	18.8	<0.001
Dim 5—Organization and guidance of nursing practice	57.2	19.1	59.2	18.0	<0.001
Dim 6—Quality and safety of nursing care	61.7	20.6	63.6	19.1	<0.001
Structure subscale	52.9	14.3	54.1	14.3	<0.001
PROCESS Component					
Dim 1—Collaboration and teamwork	65.5	12.6	69.0	13.0	<0.001
Dim 2—Strategies for ensuring quality in professional practice	58.2	16.6	57.5	16.8	0.605
Dim 3—Autonomous practices in professional practice	70.8	13.6	64.3	14.1	<0.001
Dim 4—Care planning, evaluation and continuity	70.5	13.6	61.1	15.0	<0.001
Dim 5—Theoretical and legal support of professional practice	72.7	14.8	69.6	15.1	<0.001
Dim 6—Interdependent practices in professional practice	45.4	17.2	51.6	16.5	<0.001
Process subscale	64.6	10.5	60.1	11.0	<0.001
OUTCOME Component					
Dim 1—Systematic assessment of nursing care and indicators	52.4	18.7	54.0	18.5	<0.001
Dim 2—Systematic assessment of nurses’ performance and supervision	42.6	19.2	45.4	19.9	<0.001
Outcome subscale	47.9	17.3	50.1	17.5	<0.001

* Wilcoxon test. Dim: Dimension; Std. Dev.: Standard Deviation.

**Table 3:** Effect of characterization variables on nursing practice environments: results of model estimation.

Variables	Pre-COVID-19		After the Fourth Critical Period of COVID-19	
	Estimate	*p*	Estimate	*p*
STRUCTURE Component				
Female	2.305	0.008	--	--
Married	--	--	−2.144	0.006
Divorced	3.309	0.028	--	--
Medicine Department	--	--	3.384	<0.001
Surgery Department	−3.267	<0.001	--	--
Psychiatry and Mental Health Department	--	--	3.516	0.024
Woman and Child Department	4.228	0.010	4.254	0.010
Areas of care for COVID-19 patients	--	--	−1.996	0.007
Time of professional practice	0.221	<0.001	0.212	<0.001
Time of professional practice in the service	−0.274	<0.001	−0.168	0.004
PROCESS Component				
Female	1.282	0.047	1.891	0.005
Medicine Department	--	--	3.102	<0.001
Surgery Department	−1.730	0.006	--	--
Emergency and Intensive Care Department	−2.885	<0.001	--	--
Psychiatry and Mental Health Department			3.044	0.010
Woman and Child Department	2.871	0.019	5.281	<0.001
OUTCOME Component				
Female	2.230	0.035	--	--
Married	−3.734	<0.001	−2.821	0.002
Age	0.178	0.004	--	--
Surgery Department	--	--	−2.132	0.039
Emergency and Intensive Care Department	−2.382	0.026	−5.144	<0.001
Areas of care for COVID-19 patients	--	--	−2.786	0.003
Time of professional practice in the service	−0.244	<0.001	--	--

**Table 4:** Effect of the characterisation variables on the dimensions of the components *Structure, Process* and *Outcome* of nursing professional practice environments: results of the models’ estimation.

Variables	Structure Subscale						Process Subscale						Outcome Subscale	
	Dimension 1 (Estimate *p*)	Dimension 2 (Estimate *p*)	Dimension 3 (Estimate *p*)	Dimension 4 (Estimate *p*)	Dimension 5 (Estimate *p*)	Dimension 6 (Estimate *p*)	Dimension 1 (Estimate *p*)	Dimension 2 (Estimate *p*)	Dimension 3 (Estimate *p*)	Dimension 4 (Estimate *p*)	Dimension 5 (Estimate *p*)	Dimension 6 (Estimate *p*)	Dimension 1 (Estimate *p*)	Dimension 2 (Estimate *p*)
Female						4.203 (<0.001)	2.910 (<0.001)	2.770 (0.007)						
Age		−0.342 (0.019)								0.232 (<0.001)		−0.501 (0.001)		0.126 (0.026)
Single		1.850 (0.046)										−2.748 (0.006)	2.866 (0.005)	
Married			−2.935 (0.002)							−2.589 (<0.001)				−3.897 (<0.001)
Divorced	4.878 (0.011)													
Bachelor’s degree		−2.091 (0.037)											2.678 (0.026)	
Medicine Department		3.884 (<0.001)			2.025 (0.031)		3.371 (<0.001)	2.295 (0.008)		2.537 (0.001)				
Surgery Department	−3.881 (<0.001)			−2.760 (0.015)					−3.383 (<0.001)		−3.354 (<0.001)		−3.630 (<0.001)	
Emergency and Intensive Care Department			−6.145 (<0.001)	−6.840 (<0.001)					−7.520 (<0.001)		−2.076 (0.035)		−5.080 (<0.001)	−4.656 (<0.001)
Psychiatry and Mental Health Department				4.951 (0.014)										
Woman and Child Department					7.227 (<0.001)	9.160 (<0.001)			3.758 (0.022)	9.306 (<0.001)		4.942 (0.009)		−5.868 (0.010)
Areas of care for COVID-19 patients		3.969 (<0.001)	2.934 (0.002)				3.546 (<0.001)					2.623 (0.002)		−3.731 (<0.001)
Specialist nurse			1.891 (0.042)						−1.953 (0.007)	−1.877 (0.019)		2.299 (0.011)		
Time of professional practice	0.263 (<0.001)	0.532 (<0.001)				0.190 (0.008)	0.018 (0.020)					0.387 (0.012)		
Time of professional practice in the service						−0.112 (<0.001)				−0.196 (<0.001)				

## Data Availability

The data that support the findings of this study are available from the corresponding author upon reasonable request.
